# Exact solutions for (1 + 1)-dimensional nonlinear dispersive modified Benjamin-Bona-Mahony equation and coupled Klein-Gordon equations

**DOI:** 10.1186/2193-1801-3-724

**Published:** 2014-12-10

**Authors:** Kamruzzaman Khan, M Ali Akbar, S M Rayhanul Islam

**Affiliations:** Department of Mathematics, Pabna University of Science & Technology, Pabna, 6600 Bangladesh; Department of Applied Mathematics, University of Rajshahi, Rajshahi, 6205 Bangladesh

**Keywords:** MSE method, NLEEs, DMBBM equation, cKG equation, Solitary wave, Exact solutions

## Abstract

**Abstract:**

In this work, recently developed modified simple equation (MSE) method is applied to find exact traveling wave solutions of nonlinear evolution equations (NLEEs). To do so, we consider the (1 + 1)-dimensional nonlinear dispersive modified Benjamin-Bona-Mahony (DMBBM) equation and coupled Klein-Gordon (cKG) equations. Two classes of explicit exact solutions–hyperbolic and trigonometric solutions of the associated equations are characterized with some free parameters. Then these exact solutions correspond to solitary waves for particular values of the parameters.

**PACS numbers:**

02.30.Jr; 02.70.Wz; 05.45.Yv; 94.05.Fg

## Introduction

The study of NLEEs, i.e., partial differential equations with time derivatives has a rich and long history, which has continued to attract attention in the last few decays. There are many examples throughout the world where NLEEs play an important role in controlling the natural systems. Because the majority of the phenomena in real world can be described by using NLEEs. NLEEs are frequently used to explain many problems of meteorology, population dynamics, fluid mechanics, plasma physics, optical fibers, biology, solid state physics, chemical kinematics, geochemistry, nanotechnology etc. By the aid of exact solutions, when they exist, the phenomena modeled by these NLEEs can be better understood. Therefore, the study of the traveling wave solutions for NLEEs plays an important role in the study of nonlinear physical phenomena.

In recent years, the exact solutions of NLEEs have been investigated by many authors who are interested in nonlinear physical phenomena. Many powerful methods have been presented by diverse group of mathematicians and physicists such as the Hirota’s bilinear transformation method (Hirota 1973) (Hirota and Satsuma 1981), the tanh-function method (Malfliet 1992; Nassar *et al*. 2011), the F-expansion method (Zhou et al. 2003), the (*G′*/*G*)-expansion method (Wang *et al*. 2008; Zayed 2010; Zayed and Al-Joudi 2010, Zayed and Gepreel 2009; Akbar *et al*. 2012a, 2012b, 2012c, 2012d; Akbar and Ali 2011a; Shehata 2010; Zayed and Al-Joudi 2010; Naher et al. 2012a, 2013; Naher and Abdullah 2012, 2013), the enhanced (*G′*/*G*)-expansion method (Khan et al. 2014, Khan and Akbar, 2014; Islam *et al*. 2014), the Exp-function method (He and Wu 2006; Bekir and Boz 2008; Akbar and Ali 2011b; Naher *et al*. 2011, 2012b; Yusufoglu 2008), the homogeneous balance method (Wang 1995; Zayed *et al*, 2004), the Adomian decomposition method (Adomian 1994), the homotopy perturbation method (Mohiud-Din 2007), the extended tanh-method (Abdou 2007; Fan 2000), the auxiliary equation method (Sirendaoreji 2004), the Jacobi elliptic function method (Ali 2011), Modified Exp-function method (He *et al*. 2012), the Modified simple equation method (Jawad et al. 2010; Zayed 2011; Zayed and Ibrahim 2012) and so on.

The purpose of this paper is to apply the MSE method to construct the exact solutions for nonlinear evolution equations in mathematical physics via the DMBBM equation and cKG equation. The DMBBM equation and cKG equation are NLEEs representing the balance of dispersion and weak nonlinearity in physical systems that generate solitary waves.

The article is prepared as follows: The MSE method, Applications, Graphical representation of some obtained solutions, Comparisons, and conclusions.

## The MSE method

Consider a general nonlinear evolution in the form
2.1

where *ℜ* is a polynomial of *u*(*x*, *y*, *z*, *t*) and its partial derivatives in which the highest order derivatives and nonlinear terms are involved. In the following, we give the main steps of this method (Jawad et al., 2010; Zayed, 2011, Zayed and Ibrahim, 2012):

Step 1. Using the traveling wave transformation
2.2

Eq. (2.1) transform to the following ODE:
2.3

where *℘* is a polynomial in *u*(*ξ*) and its derivatives, while , and so on.

Step 2. We suppose that Eq.(2.3) has the formal solution
2.4

where *β*_*k*_ are arbitrary constants to be determined, such that *β*_*N*_ ≠ 0, and Φ(*ξ*) is an unknown function to be determined later.

Step 3. We determine the positive integer *N* in Eq. (2.4) by considering the homogeneous balance between the highest order derivatives and the nonlinear terms in Eq. (2.3).

Step 4. We substitute (2.4) into (2.3), we calculate all the necessary derivatives *u*′ , *u*″ , ⋯ and then we account the function Φ(*ξ*). As a result of this substitution, we get a polynomial of Φ′(*ξ*)/Φ(*ξ*) and its derivatives. In this polynomial, we equate with zero all the coefficients of Φ^- *i*^(*ξ*), where *i* = 0, 1, 2, ⋯. This operation yields a system of equations which can be solved to find *β*_*k*_ and Φ(*ξ*). Consequently, we can get the exact solutions of Eq. (2.1).

## Applications

### The (1 + 1)-dimensional nonlinear dispersive modified Benjamin-Bona

**Mahony equation:** In this section, we will apply the modified simple equation method to find the exact solutions and then the solitary wave solutions of (1 + 1)-dimensional nonlinear DMBBM equation,
3.1

where *α* is a nonzero constant. This equation was first derived to describe an approximation for surface long waves in nonlinear dispersive media. It can also characterize the hydro magnetic waves in cold plasma, acoustic waves in inharmonic crystals and acoustic gravity waves in compressible fluids (Yusufoglu 2008; Zayed and Al-Joudi 2010).

The traveling wave transformation is
3.2

Using traveling wave Eq. (3.2), Eq. (3.1) transforms into the following ODE
3.3

Integrating with respect to *ξ* choosing constant of integration as zero, we obtain the following ODE
3.4

Now balancing the highest order derivative *u*″ and non-linear term *u*^3^, we get

3*N* = *N* + 2, which gives *N* = 1

Now for *N* = 1,  becomes
3.5

where *β*_0_ and *β*_1_ are constants to be determined such that *β*_1_ ≠ 0, while *ψ*(*ξ*) is an unknown function to be determined. It is easy to see that
3.63.7

Now substituting the values of *u*″, *u*, *u*^3^ in equation (3.3) and then equating the coefficients of Φ^0^, Φ^- 1^, Φ^- 2^, Φ^- 3^ to zero, we respectively obtain
3.83.93.103.11

Solving Eq. (3.8), we get


Solving Eq. (3.11), we get

 and *β*_1_ ≠ 0

**Case I:** when *β*_0_ = 0 solving Eqs. (3.9), and (3.10) we get trivial solution. So this case is rejected.

**Case II:** when , Eqs. (3.9) and (3.10) yields
3.12

where .

Integrating, Eq. (3.12) with respect to *ξ*, we obtain
3.13

From Eqs. (3.13) and (3.10), we obtain
3.14

Therefore, upon integration, we obtain
3.15

where *c*_1_ and *c*_2_ are arbitrary constants.

Substituting the values of Φ and Φ′ into Eq. (3.5), we obtain the following exact solution,
3.16

Putting the values of *β*_0_, *β*_1_, *l* and simplifying, we obtain
3.17

Since *c*_1_ and *c*_2_ are arbitrarily constants, consequently, if we set *c*_1_ = - 2 *c*_2_(1 - *ω*) and , Eq. (3.17) reduces to the following traveling wave solution:
3.18

Again setting *c*_1_ = 2 *c*_2_(1 - *ω*) and if , Eq. (3.17) reduces to the following singular traveling wave solutions:
3.19

If , Eqs. (3.18) and (3.19) yields the following periodic solutions:
3.20

and
3.21

**Remark 1:** From solutions (3.18)-(3.21) we conclude that *ω* ≠ 1.

### The coupled Klein-Gordon equation

Now we will bring to bear the MSE method to find exact solutions and then the solitary wave solutions to the cKG Equation in the form,
3.22

where
3.23

The traveling wave Eq. (3.23) reduces Eqs. (3.22) into the following ODEs
3.243.25

By integrating Eq. (3.25) with respect to *ξ*, and neglecting the constant of integration we obtain
3.26

Substituting Eq. (3.26) into Eq. (3.24) we get,
3.27

Balancing the highest order derivative *u*″ and nonlinear term *u*^3^ from Eq. (3.27), we obtain *N* = 1

Now for *N* = 1, Eq. (2.4) becomes
3.28

where *β*_0_ and *β*_1_ are constants to be determined such that *β*_1_ ≠ 0, while Φ(*ξ*) is an unknown function to be determined. It is easy to see that
3.293.30

Now substituting the values of *u*″, *u*, *u*^3^ in Eq. (3.27) and then equating the coefficients of Φ^0^, Φ^- 1^, Φ^- 2^, Φ^- 3^ to zero, we respectively obtain
3.313.323.333.34

Solving Eq. (3.31), we get


Solving Eq. (3.34), we get


**Case-I**: When *β*_0_ = 0, Eq. (3.32) and (3.33) yields a trivial solution. So this case is rejected.

**Case-II**: When , Eqs. (3.32) and (3.33) yields,
3.35

Integrating, Eq. (3.35) with respect to *ξ*, we obtain
3.36

From Eqs. (3.36) and (3.32), we obtain
3.37

Therefore, integration Eq. (3.37), we obtain
3.38

where *c*_1_ and *c*_2_ are constants of integration.

Substituting the values of Φ and Φ′ into Eq. (3.28), we obtain the following exact solution,
3.39

Putting the values of *β*_0_, *β*_1_ into Eq. (3.39) and then simplifying, we obtain
3.40

We can freely choose the constants *c*_1_ and *c*_2_. Therefore, if we set , Eq. (3.40) reduces to:
3.41

Again, if we set , Eq. (3.40) reduces to:
3.42

Using hyperbolic identities, in trigonometric form Eqs. (3.41) and (3.42) can be written as follows:
3.433.44

Now applying Eqs. (3.41)-(3.44) into Eq. (3.26), we get
3.453.463.473.48

**Remark 2:** From solutions (3.41)-(3.48) we conclude that *ω* ≠ ± 1.

## Graphical representation of some obtained solutions

In this section, we put forth to illustrate the three-dimensional and two-dimensional structure of the determined solutions of the studied NLEEs, to visualize the inner mechanism of them.

**Figure 1:**
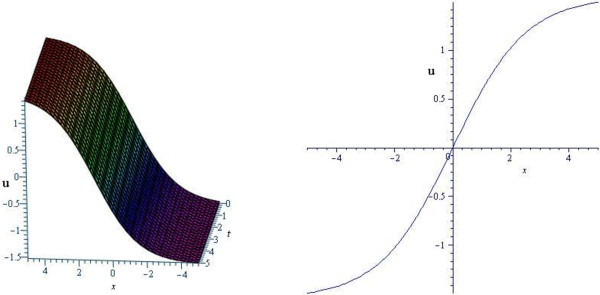
**Kink (topological soliton) profile of DMBBM equation for** ***ω*** **=0.20,** ***α*** **=1.** (Only shows the shape of (3.18), The left figure shows the 3-D plot and the right figure shows the 2-D plot for *t* = 0.

**Figure 2:**
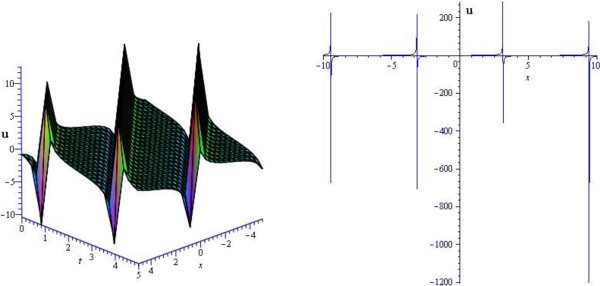
**Periodic graph of DMBBM equation for** ***ω*** **=2,** ***α*** **=3.** (Only shows the shape of (3.20)), The left figure shows the 3-D plot and the right figure shows the 2-D plot for *t* = 0.

**Figure 3:**
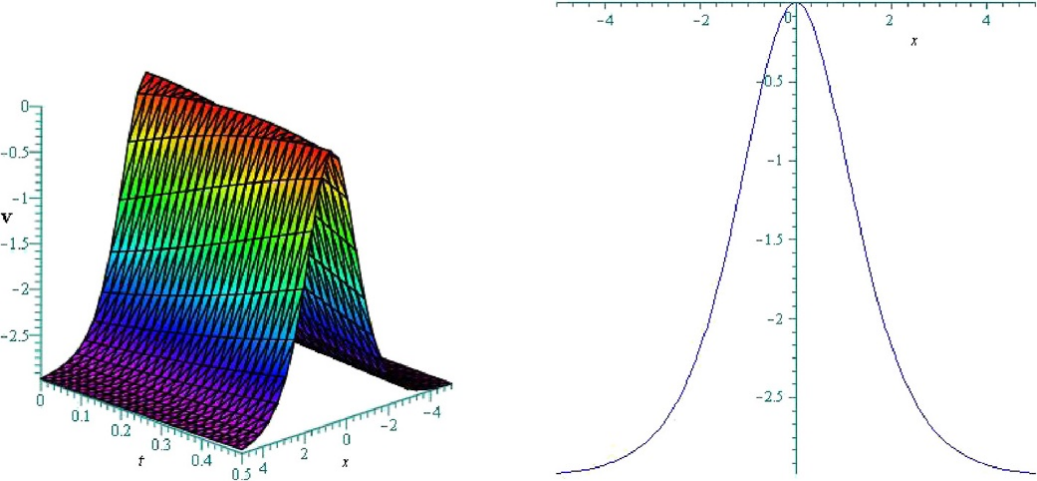
**Bell (non- topological soliton) profile of cKG equation for** ***ω*** **=1.50.** (Only shows the shape of solution (3.46)), The left figure shows the 3-D plot and the right figure shows the 2-D plot for *t* = 0.

**Figure 4:**
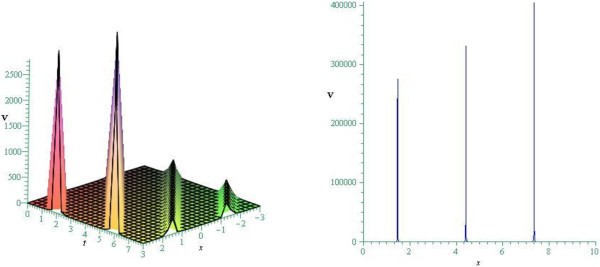
**Periodic profile of cKG equation for** ***ω*** **=0.75.** (Only shows the shape of (3.48)), The left figure shows the 3-D plot and the right figure shows the 2-D plot for *t* = 0.

Figure 1 and Figure 2 represent the shape of solutions (3.18) and (3.20) of DMBBM equation. On the other hand, Figure 3 and Figure 4 show the profile of solutions (3.46) and (3.48) of cKG equation.

## Comparisons

**With extended** (*G′/G*)**-expansion method:**

Zayed and Al-Joudi (2010) investigated exact solutions of the traveling wave solutions of the DMBBM equation by using the extended (*G′/G*)-expansion method and obtained six solutions. On the contrary by using the MSE method in this article we obtained eight solutions. However, Some of the solutions obtained by Zayed and Al-Joudi (2010) coincide with our solutions. If we set *ω* = 1 + 2*μ* in our solutions (3.18) and (3.19), we conclude that our results coincide to the solution (3.9) obtained by Zayed and Al-Joudi (2010) for *A* ≠ 0, *B* = 0, *μ* < 0, *σ* = ± 1 and *A* = 0, *B* ≠ 0, *μ* < 0, *σ* = ± 1 respectively. Similarly, solutions (3.20) and (3.21) obtained in this article correspond to the solution (3.12) obtained by Zayed and Al-Joudi (2010) for *A* ≠ 0, *B* = 0, *μ* > 0, *σ* = ± 1 and *A* = 0, *B* ≠ 0, *μ* > 0, *σ* = ± 1 respectively.

Moreover, Zayed and Al-Joudi (2010) used the symbolic computation software such as Maple or Mathematica to facilitate the calculation of the algebraic equations occurred in the solution procedure. Without symbolic computation software even it is impossible to get the solutions of the complicated algebraic equations. In addition, Zayed and Al-Joudi (2010) used the solutions of an auxiliary equation *G*″(*ξ*) + *μG*(*ξ*) = 0 to find exact traveling wave solutions of NLEEs. On the other hand it is worth mentioning that the exact solutions of the studied NLEEs have been achieved in this article without using any symbolic computations software, since the computations are very simple and easy. Similarly for any nonlinear evolution equation it can be shown that the MSE method is much easier than other methods. Furthermore, auxiliary equations are unnecessary to solve NLEEs by means of MSE methods, i.e., there exists no predefined functions or equations in MSE method.

## Conclusions

This study shows that the MSE method is quite efficient and practically well suited for use to find exact traveling wave solutions of the DMBBM equation and cKG equation. We have obtained exact solutions of these equations in terms of the hyperbolic and trigonometric functions. This study also shows that the procedure is simple, direct and constructive. The reliability of the method and the reduction in the size of computational domain give this method a wider applicability. We conclude that the studied method can be used for many other NLEEs in mathematical physics and engineering fields.
